# 2-((1*E*)-1-{2-[(2*Z*)-3,4-Diphenyl-2,3-di­hydro-1,3-thia­zol-2-yl­idene]hydrazin-1-yl­idene}eth­yl)pyridin-1-ium bromide monohydrate

**DOI:** 10.1107/S1600536814006229

**Published:** 2014-03-26

**Authors:** Mehmet Akkurt, Joel T. Mague, Shaaban K. Mohamed, Alaa A. Hassan, Mustafa R. Albayati

**Affiliations:** a Department of Physics, Faculty of Sciences, Erciyes University, 38039 Kayseri, Turkey; b Department of Chemistry, Tulane University, New Orleans, LA 70118, USA; c Chemistry and Environmental Division, Manchester Metropolitan University, Manchester M1 5GD, England; d Chemistry Department, Faculty of Science, Minia University, 61519 El-Minia, Egypt; e Kirkuk University, College of Science, Department of Chemistry, Kirkuk, Iraq

## Abstract

In the title compound, C_22_H_19_N_4_S^+^·Br^−^·H_2_O, the dihedral angles between the phenyl groups and the mean plane of the thia­zolyl­idene ring are 34.69 (13) and 64.27 (13)°, respectively, while that between the thia­zolyl­idene and pyridinium rings is 14.73 (13)°. In the crystal, zigzag chains of alternating bromide ions and water mol­ecules associate through O—H⋯Br inter­actions run in channels approximately parallel to the *b* axis. These chains help form parallel chains of cations through N—H⋯O, C—H⋯N and C—H⋯Br hydrogen bonds.

## Related literature   

For the synthesis of thia­zoles see: Zambon *et al.* (2008 ▶); Franklin *et al.* (2008 ▶); Karegoudar *et al.* (2008 ▶); Ochiai *et al.* (2003 ▶). For the biological significance of thia­zole scaffold compounds, see: Masquelin & Obrecht (2001 ▶); Hirai *et al.* (1980 ▶); Ali & El–Kazak (2010 ▶); Andreani *et al.* (1996 ▶, 2008 ▶); Budriesi *et al.* (2008 ▶); Walczynski *et al.* (2005 ▶). For similar structures, see: Mague *et al.* (2014 ▶); Mohamed *et al.* (2013*a* ▶,*b* ▶).
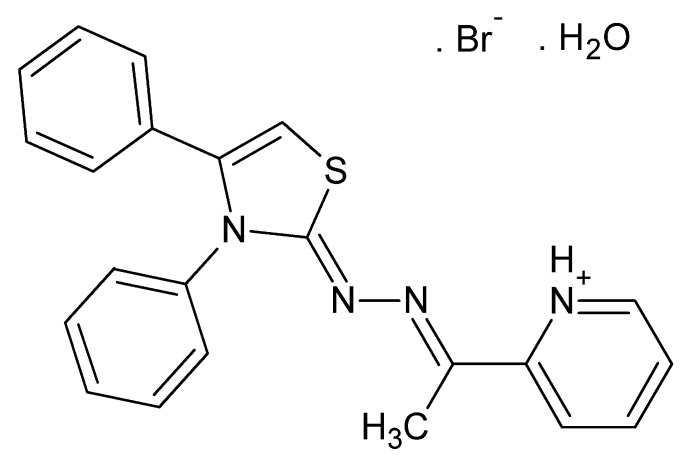


## Experimental   

### 

#### Crystal data  

C_22_H_19_N_4_S^+^·Br^−^·H_2_O
*M* *_r_* = 469.40
Orthorhombic, 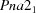

*a* = 21.8890 (17) Å
*b* = 5.7384 (4) Å
*c* = 16.6941 (13) Å
*V* = 2096.9 (3) Å^3^
*Z* = 4
Mo *K*α radiation
μ = 2.08 mm^−1^
*T* = 150 K
0.19 × 0.08 × 0.06 mm

#### Data collection  

Bruker SMART APEX CCD diffractometer
Absorption correction: multi-scan (*SADABS*; Bruker, 2013 ▶) *T*_min_ = 0.69, *T*_max_ = 0.89
35645 measured reflections
5394 independent reflections
4943 reflections with *I* > 2σ(*I*)
*R*_int_ = 0.046

#### Refinement  

*R*[*F* ^2^ > 2σ(*F* ^2^)] = 0.027
*wR*(*F* ^2^) = 0.060
*S* = 1.05
5394 reflections
263 parameters
71 restraints
H-atom parameters constrained
Δρ_max_ = 0.60 e Å^−3^
Δρ_min_ = −0.18 e Å^−3^
Absolute structure: Flack parameter determined using 2220 quotients [(*I* ^+^)−(*I* ^−^)]/[(*I* ^+^)+(*I* ^−^)] (Parsons *et al.*, 2013 ▶)
Absolute structure parameter: 0.011 (4)

### 

Data collection: *APEX2* (Bruker, 2013 ▶); cell refinement: *SAINT* (Bruker, 2013 ▶); data reduction: *SAINT*; program(s) used to solve structure: *SHELXTL* (Sheldrick, 2008 ▶); program(s) used to refine structure: *SHELXTL*; molecular graphics: *DIAMOND* (Brandenburg & Putz, 2012 ▶); software used to prepare material for publication: *SHELXTL*.

## Supplementary Material

Crystal structure: contains datablock(s) global, I. DOI: 10.1107/S1600536814006229/xu5779sup1.cif

Structure factors: contains datablock(s) I. DOI: 10.1107/S1600536814006229/xu5779Isup2.hkl

Supporting information file. DOI: 10.1107/S1600536814006229/xu5779Isup3.cml

CCDC reference: 992782

Additional supporting information:  crystallographic information; 3D view; checkCIF report

## Figures and Tables

**Table 1:** Hydrogen-bond geometry (Å, °)

*D*—H⋯*A*	*D*—H	H⋯*A*	*D*⋯*A*	*D*—H⋯*A*
O1—H1*A*⋯Br1	0.84	2.45	3.276 (2)	170
O1—H1*B*⋯Br1^i^	0.84	2.49	3.330 (2)	174
N4—H4⋯O1^ii^	0.89	1.98	2.729 (3)	141
C15—H15⋯N2^i^	0.95	2.62	3.566 (4)	178
C20—H20⋯Br1^iii^	0.95	2.72	3.645 (3)	166

Symmetry codes: (i) 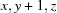; (ii) 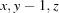; (iii) 
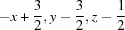
.

**Fig. 1.**
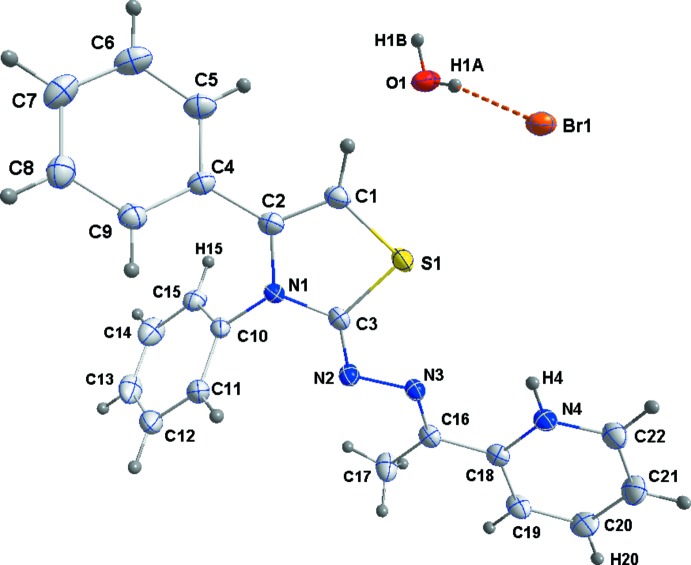
Perspective view of the asymmetric unit showing one of the O—H···Br interactions as a dotted line. Displacement ellipsoids are drawn at the 50% probability level.

**Fig. 2.**
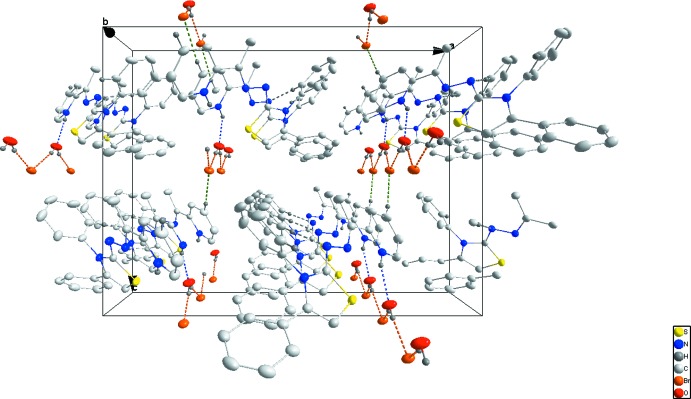
Packing viewed down the b axis showing the interionic interactions as dotted lines (O—H···Br, orange; N—H···O, blue; C—H···Br, green; C—H···N, grey.

## supplementary crystallographic information

### 1. Comment

Several methods for the synthesis of thiazole derivatives have been developed (Zambon *et al.*, 2008; Franklin *et al.*, 2008; Karegoudar *et al*., 2008) with the most widely used method being the Hantzsch's synthesis utilizing thioamides and α–halocarbonyl compounds as the starting materials (Ochiai *et al.*, 2003). 1,3–Thiazole scaffold compounds are present in many pharmacologically active substances (Masquelin & Obrecht, 2001). They have found to possess strong anti–inflammatory (Hirai *et al.*, 1980), antimicrobial (Ali & El–Kazak, 2010), antitumor (Andreani *et al.*, 2008) and selective cardiodepressant activities (Budriesi *et al.*, 2008). Other compounds containing the thiazole ring have been reported as being histamine H3 antagonists (Walczynski *et al.*, 2005) and herbicidals (Andreani *et al.*, 1996). In view of these findings and as part of our efforts (Mague *et al.*, 2014; Mohamed *et al.*, 2013*a*,*b*) to identify new candidates that may be of value in designing new and potent antimicrobial agents we report the synthesis and crystal structure of the title compound.

In the title compound (I, Fig. 1), the dihedral angle between the S1/N1C1–C3 thiazolylidene and N4/C18–C22 pyridinium rings is 14.73 (13)° while that between the phenyl groups C4–C9 and C10–C15 and the mean plane of the thiazolylidene ring are, respectively, 34.69 (13) and 64.27 (13)°. The N1–C3–N2–N3, C3–N2–N3–C16, N2–N3–C16–C17, N2–N3–C16–C18 and N3–C16–C18–C19 torsion angles are 174.4 (2), -172.8 (2), 5.7 (4), -174.3 (2) and 170.7 (3) °, respectively. The bond lengths and bond angles in (I) are normal and comparable to those previously reported for similar structures (Mague *et al.*, 2014; Mohamed *et al.*, 2013*a*,*b*).

In the crystal, zigzag chains of alternating bromide ions and water molecules associated through O—H···Br interactions run in channels approximately parallel to the *b* axis. These chains help form parallel chains of cations through N—H···O, C—H···N and C—H···Br hydrogen bonds (Fig. 2 and Table 1).

### 2. Experimental

The title compound has been prepared according to our reported method (Mohamed *et al.*, 2013*b*). Orange crystals suitable for X-ray diffraction (m.p.: 507 K) have been obtained by crystallization of the crude product (I) from ethanol.

### 3. Refinement

H-atoms attached to carbon were placed in calculated positions (C—H = 0.95 - 0.98 Å) while those attached to nitrogen and oxygen were placed in locations derived from a difference map and their coordinates adjusted to give N—H = 0.89 and O—H = 0.84 Å. All were included as riding contributions with isotropic displacement parameters 1.2 - 1.5 times those of the attached atoms.

### Crystal data

**Table:**

C_22_H_19_N_4_S^+^·Br^−^·H_2_O	*F*(000) = 960
*M**_r_* = 469.40	*D*_x_ = 1.487 Mg m^−^^3^
Orthorhombic, *P**n**a*2_1_	Mo *K*α radiation, λ = 0.71073 Å
Hall symbol: P 2c -2n	Cell parameters from 9578 reflections
*a* = 21.8890 (17) Å	θ = 2.2–28.6°
*b* = 5.7384 (4) Å	µ = 2.08 mm^−^^1^
*c* = 16.6941 (13) Å	*T* = 150 K
*V* = 2096.9 (3) Å^3^	Column, orange
*Z* = 4	0.19 × 0.08 × 0.06 mm

### Data collection

**Table:**

Bruker SMART APEX CCD diffractometer	5394 independent reflections
Radiation source: fine-focus sealed tube	4943 reflections with *I* > 2σ(*I*)
Graphite monochromator	*R*_int_ = 0.046
Detector resolution: 8.3660 pixels mm^-1^	θ_max_ = 28.9°, θ_min_ = 1.9°
φ and ω scans	*h* = −29→29
Absorption correction: multi-scan (*SADABS*; Bruker, 2013)	*k* = −7→7
*T*_min_ = 0.69, *T*_max_ = 0.89	*l* = −22→21
35645 measured reflections	

### Refinement

**Table:**

Refinement on *F*^2^	Hydrogen site location: inferred from neighbouring sites
Least-squares matrix: full	H-atom parameters constrained
*R*[*F*^2^ > 2σ(*F*^2^)] = 0.027	*w* = 1/[σ^2^(*F*_o_^2^) + (0.0251*P*)^2^] where *P* = (*F*_o_^2^ + 2*F*_c_^2^)/3
*wR*(*F*^2^) = 0.060	(Δ/σ)_max_ = 0.001
*S* = 1.05	Δρ_max_ = 0.60 e Å^−^^3^
5394 reflections	Δρ_min_ = −0.18 e Å^−^^3^
263 parameters	Absolute structure: Flack parameter determined using 2220 quotients [(*I*^+^)-(*I*^-^)]/[(*I*^+^)+(*I*^-^)] (Parsons *et al.*, 2013)
71 restraints	Absolute structure parameter: 0.011 (4)

### Special details

**Table:**

Geometry. Bond distances, angles *etc*. have been calculated using the rounded fractional coordinates. All su's are estimated from the variances of the (full) variance-covariance matrix. The cell e.s.d.'s are taken into account in the estimation of distances, angles and torsion angles
Refinement. Refinement on *F*^2^ for ALL reflections except those flagged by the user for potential systematic errors. Weighted *R*-factors *wR* and all goodnesses of fit *S* are based on *F*^2^, conventional *R*-factors *R* are based on *F*, with *F* set to zero for negative *F*^2^. The observed criterion of *F*^2^ > σ(*F*^2^) is used only for calculating -*R*-factor-obs *etc*. and is not relevant to the choice of reflections for refinement. *R*-factors based on *F*^2^ are statistically about twice as large as those based on *F*, and *R*-factors based on ALL data will be even larger.

### Fractional atomic coordinates and isotropic or equivalent isotropic displacement parameters (Å^2^)

**Table:**

	*x*	*y*	*z*	*U*_iso_*/*U*_eq_	
S1	0.61850 (3)	0.09593 (11)	0.85092 (4)	0.0229 (2)	
N1	0.51800 (10)	0.2131 (3)	0.78160 (14)	0.0192 (6)	
N2	0.57004 (10)	−0.0860 (4)	0.71385 (14)	0.0217 (6)	
N3	0.61924 (10)	−0.2324 (4)	0.72594 (15)	0.0205 (6)	
N4	0.71202 (10)	−0.5216 (4)	0.75415 (13)	0.0213 (6)	
C1	0.57446 (12)	0.3189 (4)	0.89084 (17)	0.0230 (8)	
C2	0.52293 (11)	0.3602 (4)	0.84921 (17)	0.0206 (7)	
C3	0.56577 (11)	0.0597 (4)	0.77396 (16)	0.0194 (7)	
C4	0.47512 (11)	0.5271 (4)	0.87249 (16)	0.0205 (7)	
C5	0.49212 (15)	0.7326 (4)	0.91199 (19)	0.0265 (8)	
C6	0.44860 (15)	0.8887 (5)	0.93869 (18)	0.0310 (9)	
C7	0.38714 (15)	0.8462 (5)	0.92659 (19)	0.0314 (9)	
C8	0.36944 (14)	0.6413 (5)	0.88887 (18)	0.0281 (8)	
C9	0.41249 (12)	0.4834 (5)	0.86216 (16)	0.0236 (8)	
C10	0.47325 (11)	0.2338 (4)	0.71884 (18)	0.0194 (7)	
C11	0.43411 (12)	0.0479 (5)	0.70314 (18)	0.0259 (8)	
C12	0.39293 (13)	0.0661 (5)	0.6403 (2)	0.0337 (10)	
C13	0.39056 (15)	0.2685 (6)	0.5947 (2)	0.0362 (10)	
C14	0.42835 (15)	0.4519 (6)	0.61191 (18)	0.0351 (10)	
C15	0.47038 (12)	0.4360 (5)	0.67431 (16)	0.0247 (8)	
C16	0.63290 (12)	−0.3691 (5)	0.66705 (16)	0.0208 (7)	
C17	0.60418 (15)	−0.3685 (6)	0.58542 (18)	0.0324 (9)	
C18	0.68144 (11)	−0.5385 (4)	0.68431 (15)	0.0195 (7)	
C19	0.69676 (14)	−0.7211 (5)	0.63304 (17)	0.0252 (8)	
C20	0.74126 (14)	−0.8816 (5)	0.65600 (18)	0.0290 (9)	
C21	0.77059 (14)	−0.8562 (6)	0.7283 (2)	0.0302 (9)	
C22	0.75536 (14)	−0.6732 (5)	0.77747 (19)	0.0265 (9)	
Br1	0.72775 (2)	0.20153 (4)	0.99633 (2)	0.0276 (1)	
O1	0.71716 (11)	0.6961 (3)	0.89932 (15)	0.0354 (7)	
H1	0.58560	0.40360	0.93750	0.0280*	
H4	0.70300	−0.40570	0.78750	0.0260*	
H5	0.53420	0.76510	0.92050	0.0320*	
H6	0.46100	1.02660	0.96560	0.0370*	
H7	0.35740	0.95570	0.94390	0.0380*	
H8	0.32720	0.60930	0.88140	0.0340*	
H9	0.39960	0.34400	0.83650	0.0280*	
H11	0.43560	−0.08920	0.73500	0.0310*	
H12	0.36630	−0.05990	0.62840	0.0400*	
H13	0.36260	0.27960	0.55140	0.0430*	
H14	0.42590	0.59080	0.58110	0.0420*	
H15	0.49680	0.56290	0.68610	0.0300*	
H17A	0.57680	−0.23420	0.58060	0.0490*	
H17B	0.58080	−0.51260	0.57790	0.0490*	
H17C	0.63620	−0.35830	0.54450	0.0490*	
H19	0.67700	−0.73610	0.58270	0.0300*	
H20	0.75130	−1.00820	0.62180	0.0350*	
H21	0.80110	−0.96440	0.74420	0.0360*	
H22	0.77530	−0.65380	0.82760	0.0320*	
H1A	0.72270	0.57880	0.92850	0.0420*	
H1B	0.71770	0.81980	0.92620	0.0420*	

### Atomic displacement parameters (Å^2^)

**Table:**

	*U*^11^	*U*^22^	*U*^33^	*U*^12^	*U*^13^	*U*^23^
S1	0.0172 (3)	0.0289 (3)	0.0225 (3)	0.0002 (3)	−0.0039 (3)	−0.0032 (3)
N1	0.0174 (11)	0.0204 (9)	0.0197 (12)	−0.0007 (8)	−0.0022 (8)	−0.0024 (8)
N2	0.0199 (11)	0.0255 (11)	0.0198 (11)	0.0030 (9)	−0.0001 (9)	−0.0031 (9)
N3	0.0171 (11)	0.0236 (10)	0.0207 (12)	−0.0002 (9)	−0.0010 (9)	−0.0009 (9)
N4	0.0211 (10)	0.0234 (10)	0.0193 (12)	0.0016 (9)	0.0014 (9)	−0.0043 (9)
C1	0.0231 (13)	0.0260 (12)	0.0198 (14)	−0.0024 (10)	−0.0016 (11)	−0.0049 (10)
C2	0.0211 (12)	0.0227 (11)	0.0180 (13)	−0.0039 (9)	0.0015 (11)	−0.0014 (10)
C3	0.0163 (12)	0.0234 (12)	0.0186 (13)	−0.0019 (10)	−0.0011 (10)	0.0005 (10)
C4	0.0255 (13)	0.0203 (11)	0.0157 (13)	−0.0004 (10)	0.0031 (10)	0.0003 (9)
C5	0.0332 (15)	0.0244 (12)	0.0218 (15)	−0.0043 (12)	0.0038 (12)	−0.0016 (10)
C6	0.0455 (18)	0.0220 (13)	0.0254 (16)	−0.0022 (12)	0.0086 (14)	−0.0026 (11)
C7	0.0420 (18)	0.0276 (14)	0.0247 (16)	0.0114 (13)	0.0082 (14)	0.0017 (11)
C8	0.0266 (14)	0.0366 (14)	0.0210 (14)	0.0056 (12)	0.0026 (12)	0.0015 (12)
C9	0.0255 (13)	0.0264 (12)	0.0190 (14)	−0.0004 (11)	−0.0011 (11)	−0.0012 (11)
C10	0.0175 (12)	0.0245 (12)	0.0162 (13)	0.0034 (10)	−0.0018 (10)	−0.0051 (10)
C11	0.0204 (13)	0.0226 (12)	0.0348 (17)	0.0012 (11)	−0.0028 (11)	−0.0047 (11)
C12	0.0239 (14)	0.0354 (16)	0.0417 (19)	0.0039 (13)	−0.0080 (13)	−0.0144 (14)
C13	0.0323 (16)	0.0530 (19)	0.0234 (16)	0.0137 (15)	−0.0102 (13)	−0.0095 (14)
C14	0.0458 (19)	0.0388 (17)	0.0206 (15)	0.0114 (15)	−0.0038 (13)	0.0031 (12)
C15	0.0284 (14)	0.0272 (13)	0.0186 (14)	0.0025 (11)	0.0008 (11)	−0.0033 (10)
C16	0.0191 (12)	0.0255 (12)	0.0178 (13)	−0.0011 (10)	0.0011 (10)	0.0002 (10)
C17	0.0326 (16)	0.0433 (16)	0.0212 (16)	0.0109 (14)	−0.0024 (12)	−0.0033 (13)
C18	0.0188 (12)	0.0229 (11)	0.0167 (12)	−0.0027 (10)	0.0036 (10)	0.0001 (10)
C19	0.0259 (14)	0.0322 (14)	0.0175 (14)	0.0014 (11)	0.0009 (11)	−0.0057 (11)
C20	0.0333 (16)	0.0281 (14)	0.0256 (16)	0.0050 (12)	0.0066 (12)	−0.0080 (12)
C21	0.0289 (16)	0.0307 (14)	0.0310 (17)	0.0088 (12)	0.0025 (12)	−0.0009 (13)
C22	0.0240 (14)	0.0310 (15)	0.0245 (16)	0.0035 (12)	−0.0009 (12)	−0.0034 (11)
Br1	0.0380 (2)	0.0227 (1)	0.0221 (1)	−0.0011 (1)	−0.0049 (1)	−0.0042 (1)
O1	0.0582 (15)	0.0222 (10)	0.0257 (12)	0.0051 (9)	−0.0093 (10)	−0.0045 (8)

### Geometric parameters (Å, º)

**Table:**

S1—C1	1.735 (3)	C14—C15	1.393 (4)
S1—C3	1.740 (3)	C16—C18	1.469 (4)
O1—H1A	0.8400	C16—C17	1.501 (4)
O1—H1B	0.8400	C18—C19	1.394 (4)
N1—C2	1.414 (3)	C19—C20	1.394 (4)
N1—C10	1.439 (4)	C20—C21	1.375 (4)
N1—C3	1.373 (3)	C21—C22	1.374 (5)
N2—C3	1.310 (3)	C1—H1	0.9500
N2—N3	1.381 (3)	C5—H5	0.9500
N3—C16	1.293 (4)	C6—H6	0.9500
N4—C18	1.348 (3)	C7—H7	0.9500
N4—C22	1.345 (4)	C8—H8	0.9500
N4—H4	0.8900	C9—H9	0.9500
C1—C2	1.346 (4)	C11—H11	0.9500
C2—C4	1.471 (3)	C12—H12	0.9500
C4—C9	1.404 (4)	C13—H13	0.9500
C4—C5	1.401 (4)	C14—H14	0.9500
C5—C6	1.382 (4)	C15—H15	0.9500
C6—C7	1.382 (5)	C17—H17C	0.9800
C7—C8	1.389 (4)	C17—H17A	0.9800
C8—C9	1.381 (4)	C17—H17B	0.9800
C10—C11	1.393 (4)	C19—H19	0.9500
C10—C15	1.379 (4)	C20—H20	0.9500
C11—C12	1.387 (4)	C21—H21	0.9500
C12—C13	1.390 (5)	C22—H22	0.9500
C13—C14	1.369 (5)		
			
C1—S1—C3	90.18 (12)	C19—C20—C21	119.8 (3)
H1A—O1—H1B	111.00	C20—C21—C22	119.5 (3)
C2—N1—C10	125.7 (2)	N4—C22—C21	119.5 (3)
C3—N1—C10	120.3 (2)	S1—C1—H1	123.00
C2—N1—C3	113.5 (2)	C2—C1—H1	123.00
N3—N2—C3	109.4 (2)	C6—C5—H5	120.00
N2—N3—C16	116.0 (2)	C4—C5—H5	120.00
C18—N4—C22	123.7 (2)	C5—C6—H6	120.00
C22—N4—H4	117.00	C7—C6—H6	120.00
C18—N4—H4	119.00	C8—C7—H7	120.00
S1—C1—C2	113.4 (2)	C6—C7—H7	120.00
C1—C2—C4	125.1 (2)	C9—C8—H8	120.00
N1—C2—C1	111.8 (2)	C7—C8—H8	120.00
N1—C2—C4	123.1 (2)	C4—C9—H9	120.00
N1—C3—N2	122.3 (2)	C8—C9—H9	120.00
S1—C3—N1	111.11 (18)	C10—C11—H11	121.00
S1—C3—N2	126.51 (19)	C12—C11—H11	121.00
C2—C4—C5	118.9 (2)	C13—C12—H12	120.00
C2—C4—C9	123.1 (2)	C11—C12—H12	120.00
C5—C4—C9	117.9 (2)	C12—C13—H13	120.00
C4—C5—C6	121.0 (3)	C14—C13—H13	120.00
C5—C6—C7	120.6 (3)	C15—C14—H14	120.00
C6—C7—C8	119.2 (3)	C13—C14—H14	120.00
C7—C8—C9	120.8 (3)	C10—C15—H15	120.00
C4—C9—C8	120.6 (3)	C14—C15—H15	120.00
C11—C10—C15	121.0 (3)	C16—C17—H17B	109.00
N1—C10—C11	119.5 (2)	C16—C17—H17C	109.00
N1—C10—C15	119.5 (2)	H17A—C17—H17B	109.00
C10—C11—C12	119.0 (3)	H17A—C17—H17C	109.00
C11—C12—C13	120.1 (3)	H17B—C17—H17C	110.00
C12—C13—C14	120.3 (3)	C16—C17—H17A	109.00
C13—C14—C15	120.4 (3)	C18—C19—H19	120.00
C10—C15—C14	119.2 (3)	C20—C19—H19	120.00
N3—C16—C17	126.3 (3)	C21—C20—H20	120.00
N3—C16—C18	114.8 (2)	C19—C20—H20	120.00
C17—C16—C18	118.9 (2)	C20—C21—H21	120.00
C16—C18—C19	123.5 (2)	C22—C21—H21	120.00
N4—C18—C19	117.8 (2)	N4—C22—H22	120.00
N4—C18—C16	118.8 (2)	C21—C22—H22	120.00
C18—C19—C20	119.7 (3)		
			
C3—S1—C1—C2	0.9 (2)	C1—C2—C4—C5	33.9 (4)
C1—S1—C3—N1	−0.31 (19)	C1—C2—C4—C9	−141.5 (3)
C1—S1—C3—N2	177.4 (2)	C2—C4—C5—C6	−176.6 (3)
C3—N1—C2—C1	1.0 (3)	C9—C4—C5—C6	−1.0 (4)
C3—N1—C2—C4	−175.7 (2)	C2—C4—C9—C8	176.7 (3)
C10—N1—C2—C1	−170.8 (2)	C5—C4—C9—C8	1.3 (4)
C10—N1—C2—C4	12.5 (4)	C4—C5—C6—C7	−0.4 (5)
C2—N1—C3—S1	−0.3 (3)	C5—C6—C7—C8	1.6 (5)
C2—N1—C3—N2	−178.1 (2)	C6—C7—C8—C9	−1.3 (5)
C10—N1—C3—S1	171.96 (17)	C7—C8—C9—C4	−0.1 (4)
C10—N1—C3—N2	−5.8 (4)	N1—C10—C11—C12	−177.3 (3)
C2—N1—C10—C11	−121.3 (3)	C15—C10—C11—C12	1.7 (4)
C2—N1—C10—C15	59.7 (4)	N1—C10—C15—C14	177.9 (3)
C3—N1—C10—C11	67.5 (3)	C11—C10—C15—C14	−1.1 (4)
C3—N1—C10—C15	−111.5 (3)	C10—C11—C12—C13	−0.8 (4)
C3—N2—N3—C16	−172.8 (2)	C11—C12—C13—C14	−0.6 (5)
N3—N2—C3—S1	8.2 (3)	C12—C13—C14—C15	1.2 (5)
N3—N2—C3—N1	−174.4 (2)	C13—C14—C15—C10	−0.4 (4)
N2—N3—C16—C17	5.7 (4)	N3—C16—C18—N4	−7.6 (4)
N2—N3—C16—C18	−174.3 (2)	N3—C16—C18—C19	170.7 (3)
C22—N4—C18—C16	177.0 (3)	C17—C16—C18—N4	172.4 (2)
C22—N4—C18—C19	−1.4 (4)	C17—C16—C18—C19	−9.3 (4)
C18—N4—C22—C21	0.5 (4)	N4—C18—C19—C20	1.7 (4)
S1—C1—C2—N1	−1.2 (3)	C16—C18—C19—C20	−176.6 (3)
S1—C1—C2—C4	175.4 (2)	C18—C19—C20—C21	−1.3 (4)
N1—C2—C4—C5	−149.9 (3)	C19—C20—C21—C22	0.4 (5)
N1—C2—C4—C9	34.8 (4)	C20—C21—C22—N4	0.0 (5)

### Hydrogen-bond geometry (Å, º)

**Table:**

*D*—H···*A*	*D*—H	H···*A*	*D*···*A*	*D*—H···*A*
O1—H1*A*···Br1	0.84	2.45	3.276 (2)	170
O1—H1*B*···Br1^i^	0.84	2.49	3.330 (2)	174
N4—H4···O1^ii^	0.89	1.98	2.729 (3)	141
C15—H15···N2^i^	0.95	2.62	3.566 (4)	178
C20—H20···Br1^iii^	0.95	2.72	3.645 (3)	166

Symmetry codes: (i) *x*, *y*+1, *z*; (ii) *x*, *y*−1, *z*; (iii) −*x*+3/2, *y*−3/2, *z*−1/2.
